# Anticonvulsant Effect of Time-Restricted Feeding in a Pilocarpine-Induced Seizure Model: Metabolic and Epigenetic Implications

**DOI:** 10.3389/fncel.2016.00007

**Published:** 2016-01-28

**Authors:** Jorge Landgrave-Gómez, Octavio Fabián Mercado-Gómez, Mario Vázquez-García, Víctor Rodríguez-Molina, Laura Córdova-Dávalos, Virginia Arriaga-Ávila, Alfredo Miranda-Martínez, Rosalinda Guevara-Guzmán

**Affiliations:** Departamento de Fisiología, Facultad de Medicina, Universidad Nacional Autónoma de MéxicoMéxico, DF, Mexico

**Keywords:** anticonvulsant, pilocarpine, AMP kinase, Akt kinase, histone 3 acetylation, beta-hydroxybutyrate, HDACs inhibition

## Abstract

A new generation of antiepileptic drugs has emerged; however, one-third of epilepsy patients do not properly respond to pharmacological treatments. The purpose of the present study was to investigate whether time-restricted feeding (TRF) has an anticonvulsant effect and whether this restrictive diet promotes changes in energy metabolism and epigenetic modifications in a pilocarpine-induced seizure model. To resolve our hypothesis, one group of rats had free access to food and water *ad libitum* (AL) and a second group underwent a TRF schedule. We used the lithium-pilocarpine model to induce *status epilepticus* (SE), and behavioral seizure monitoring was analyzed. Additionally, an electroencephalography (EEG) recording was performed to verify the effect of TRF on cortical electrical activity after a pilocarpine injection. For biochemical analysis, animals were sacrificed 24 h after SE and hippocampal homogenates were used to evaluate the proteins related to metabolism and chromatin structure. Our results showed that TRF had an anticonvulsant effect as measured by the prolonged latency of forelimb clonus seizure, a decrease in the seizure severity score and fewer animals reaching SE. Additionally, the power of the late phase EEG recordings in the AL group was significantly higher than the TRF group. Moreover, we found that TRF is capable of inducing alterations in signaling pathways that regulate energy metabolism, including an increase in the phosphorylation of AMP dependent kinase (AMPK) and a decrease in the phosphorylation of Akt kinase. Furthermore, we found that TRF was able to significantly increase the beta hydroxybutyrate (β-HB) concentration, an endogenous inhibitor of histone deacetylases (HDACs). Finally, we found a significant decrease in HDAC activity as well as an increase in acetylation on histone 3 (H3) in hippocampal homogenates from the TRF group. These findings suggest that alterations in energy metabolism and the increase in β-HB mediated by TRF may inhibit HDAC activity, thus increasing histone acetylation and producing changes in the chromatin structure, which likely facilitates the transcription of a subset of genes that confer anticonvulsant activity.

## Introduction

Epilepsy is the third most common chronic brain disorder. It affects 50 million people worldwide (Aroniadou-Anderjaska et al., 2008). Although a new generation of antiepileptic drugs has emerged, approximately 30% of epilepsy patients do not respond to classical pharmacological treatment (Löscher et al., 2013). For this reason, it is important to find new alternatives to complement pharmacological therapy in drug-resistant patients. To date, a variety of reports suggest that some metabolism-based therapies, such as ketogenic diet (KD) or calorie restricted (CR) diets, have an anticonvulsant effect (Bough et al., 2003; Stafstrom and Rho, 2012). Recently, it has been suggested that the beneficial effect of these diets may be produced by means of a metabolic shift involving the activation of AMP-activated protein kinase (AMPK), inhibition of the mammalian target of rapamycin (mTOR) and overproduction of ketone bodies (Wong, 2010; McDaniel et al., 2011; Yuen and Sander, 2014).

Time-restricted feeding (TRF) is a nutritional challenge that limits food availability to a brief time during the waking phase in mammals (Belet and Sassone-Corsi, 2010). This restrictive model induces an increase in free fatty acids (FFA) before feeding and an increase in peroxisomal markers, such as PPARα and PPARγ (Rivera-Zavala et al., 2011), suggesting that it may modulate a global metabolic shift that resembles the effects of other metabolism-based therapies.

On the other hand, environmental inputs, such as nutrition, are able to alter cell metabolism. In this sense, functional links between metabolism and epigenetic control are beginning to emerge (Sassone-Corsi, 2013). The regulation of gene expression by epigenetic modifications can occur through a variety of means. To date, the best characterized include DNA methylation, non-coding RNAs and histone posttranslational modifications (Hullar and Fu, 2014).

Histone posttranslational modifications, such as acetylation, occur at specific lysine residues and have been correlated with transcriptional activation (Sassone-Corsi, 2013). Histone deacetylases (HDACs) are enzymes that elicit the induction of repressive chromatin using specific metabolites, such as nicotinamide adenine dinucleotide (NAD^+^), whose availability dictates the efficacy of the enzymatic reaction (Katada et al., 2012). Interestingly, it has recently been shown that β-hydroxybutyrate (β-HB), a ketone body produced during fasting or starvation conditions, act as an endogenous inhibitor of HDACs, thus linking metabolism with gene expression (Shimazu et al., 2013).

In spite of these findings, there are no reports showing that TRF may produce beneficial effects, such as those of KD and CR, in an acute seizure model. For this reason, the purpose of this study was to determine whether TRF induces a metabolic shift by activating the energy sensor AMPK, inhibiting the Akt signaling pathway and producing epigenetic modifications that are capable of diminishing seizure susceptibility. Here, we report that TRF had anticonvulsant effects observed as prolonged latency to first seizure, a decrease in the seizure score, and a diminished number of animals that reached *status epilepticus* (SE). Additionally, a reduction in the power of the late phase electroencephalography (EEG) recordings in the TRF group was significantly greater than that in the AL group. Furthermore, TRF produced an increase in the β-HB concentration, activation of AMPK, inhibition of Akt kinase and increased histone 3 (H3) acetylation. These findings suggest that activation of the AMPK signaling pathway together with an increase in ketone bodies could mediate the acetylation of H3, thus contributing to the transcription of a subset of genes conferring anticonvulsant activity.

## Materials and Methods

### Time-Restricted Feeding Schedule and Pilocarpine-Induced Seizure Model

**Figure 1:**
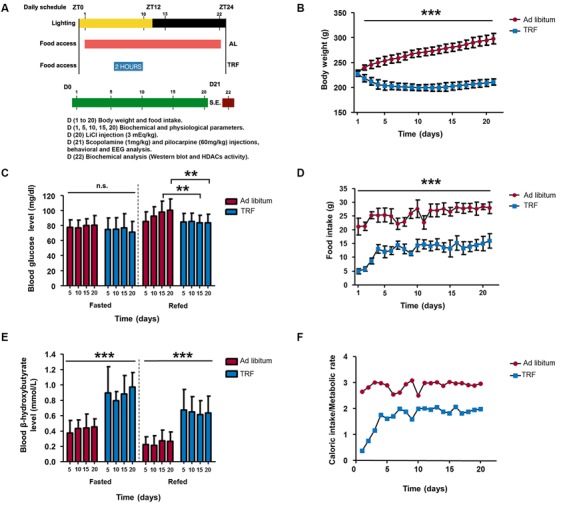
**Influence of the time-restricted feeding (TRF) model on body weight, food intake, glucose, and β-hydroxybutyrate in the fasted/refed state and the ratio among kilocalorie consumption and basal metabolic rate (BMR) in rats. (A)** Schematic representation of the experimental procedure of the dietary schedule and *status epilepticus* (SE) induction. Body weight of TRF rats showed a significant decrease at all time points measured **(B)**; moreover, there is a reduction in food intake and the ratio of caloric intake/metabolic rate compared with that of *ad libitum* (AL)-fed animals **(D,F)**. Regarding biochemical parameters, the blood glucose concentration showed no significant difference in the fasted state; however, AL-fed rats showed an inability to metabolize glucose at 15 and 20 days, which does not occur in TRF animals **(C)**. Interestingly, the blood β-hydroxybutyrate concentration was high during the TRF schedule and it was maintained even in the refed state, even though it is lower than during the fasted state **(E)**. Data are expressed as the mean ± SD from each determination (*n* = 30, **p < 0.01; ***p < 0.001).

Ninety-five young adult male Wistar rats (8 weeks of age) weighing approximately 220 g obtained from Harlan laboratories (USA) were used and maintained under constant temperature conditions (25°C) and a 12 h light/12 h dark cycle. Animals were fed with a standard diet of Lab Diet Rodent Laboratory Diet 5001 pellets (PMI Nutrition International, Inc., Brentwood MO) and water *ad libitum* (AL, control and pilocarpine groups) or underwent the time-restricted feeding (TRF and TRF plus pilocarpine groups) schedule described by Rivera-Zavala et al. (2011). Briefly, TRF consisted of allowing rats to feed freely for only 2 h daily for 20 days, and after this period of time, we proceeded to perform the acute seizure model at day 21. For the acute seizure model, we chose the lithium-pilocarpine model because it is one of the most widely used models to induce SE and is an excellent model that resembles human temporal lobe epilepsy (Lemos and Cavalheiro, 1995). Animals were first injected with lithium-chloride (3 mEq/kg, i.p.) at day 20; 18 h later, animals received a scopolamine methyl nitrate injection (1 mg/kg, s.c.) 30 min prior to minimize the peripheral cholinergic effects of pilocarpine. Pilocarpine was administered (60 mg/kg, s.c.) to induce SE, and the latter was maintained for 90 min; immediately afterwards, animals received an injection of diazepam (Valium^2122^, 5 mg/kg i.m.) to stop the seizures. The same procedure was performed for control animals that did not receive a pilocarpine injection but rather received a saline (0.9%) injection (Figure 1A). It is important to mention that SE was induced after 6 h of fasting in AL-pilocarpine rats and ≈ 22 h fasting in TRF-pilocarpine rats to avoid any changes in metabolism; both AL- and TRF-pilocarpine animals received a saline solution injection to avoid dehydration. Twenty-four hours after the pilocarpine injection, experimental animals were sacrificed with an overdose of sodium pentobarbital (26 mg/kg) to perform biochemical analyses. All experiments from the present study were approved by the Ethical Committee of the School of Medicine at UNAM following all of their statements to minimize animal suffering.

### Physical/Biochemical, Food Intake, Glucose Tolerance, Brain and Liver Weight Measurements

The food intake and body weight from each animal were monitored daily, and both blood glucose and the β-HB concentration were measured using a digital monitor system (Optium Xceed glucometer, Abbott USA) with specific glucose and β-HB strips at 5, 10, 15 and 20 days. For blood sampling, animals from the AL group were fasted for 6 h before measurement and the TRF group was measured before feeding with chow (≈ 22 h fasting). For glucose tolerance, we performed an additional experiment consisting of measuring the blood glucose in fasted rats, and then, animals were injected intraperitoneally with a glucose solution (refed rats, 1 g/kg body weight) and their blood glucose was measured again after 60 min. The same experimental procedure was carried out for the β-HB measurement to verify whether the β-HB levels remained or decayed after receiving a glucose injection. In addition, we calculated the ratio of the daily caloric intake (kcal/day) consumed for each animal from the AL and TRF groups and divided by basal metabolic rate (BMR) using an allometric scaling formula (BMR = 70M^0.75^, where M means mass) to verify whether the TRF schedule could have an effect on growth in animals. Finally, the brains and livers from the AL and TRF groups were perfused with saline, dissected and weighed to examine whether dietary restriction could affect the size of each organ.

### Latency to the First Seizure, Seizure Score and Number of Animals that Reached *Status Epilepticus*

After administration of pilocarpine, animals were continuously video monitored for seizure activity for at least 5 h by an investigator-blinded to the treatment who then scored the behavioral seizures. Latency to the first seizure was measured in minutes until the first forelimb clonus appeared, and the seizure score and the number of rats that reached SE were also analyzed. The behavioral seizures induced by pilocarpine were scored according to a modified version of the Racine scale (Bough et al., 2002). The latency to first seizure, seizure severity score, and individuals with SE were averaged across animals in each group.

### Western Blot Analysis

Experimental animals were sacrificed with an overdose of anesthesia and rat brains were dissected as quickly as possible. Immediately afterwards, hippocampi were obtained and fractioned into cytoplasmic and nuclear extracts using a subcellular fractionation kit (Thermo Scientific, USA) following the manufacturer’s instructions. Protein extracts were quantified by a BCA assay kit (Pierce, USA), and 60 μg of protein was loaded on 12 or 15% SDS-PAGE gels. Proteins were transferred to a PVDF membrane in a semi-dry electrophoretic transfer system (BioRad, USA). Then, membranes were rinsed with Tris-buffered saline (TBS) and blocked with a solution containing 5% non-fat dry milk in TBS-Tween 20 0.1% (TBST) overnight at 4°C. Blots were probed with rabbit polyclonal anti-pAMPK (Thr172; 1:1000, Cell Signaling Technology, AB 2535), polyclonal anti-pAkt (Ser473; 1:1000 Cell Signaling Technology, AB 4060) and the H3 acetylation at lysine residues 9 and 14 (H3K9ac and H3K14ac; 1:1000, Cell Signaling Technology, AB 9649 and AB 7627, respectively) in TBST overnight at 4°C. After three rinses with TBST for 5 min each, membranes were incubated with a goat anti-rabbit IgG secondary antibody (1:2500 Cell Signaling Technology, AB7074) for 2 h at room temperature followed by three rinses with TBST for 5 min each. The band signal was detected by a chemiluminescence kit (Millipore, USA) on Amersham Hyperfilm. For loading controls, rabbit polyclonal antibodies against total AMPK, Akt, and H3 (1:1000, Cell Signaling Technology, AB 5831, AB 4691 and AB 4499, respectively) were used. Densitometry measurements of the detected bands images were taken with a CCD camera (DNR Bio-Imaging Systems), and the analysis was carried out with MCID image analysis software (InterFocus Imaging Ltd., Cambridge, UK).

### Histone Deacetylase Activity Assay

Protein nuclear extracts from hippocampal homogenates were quantified by a BCA assay kit (Pierce, USA), and 80 μg of protein was used to measure HDAC activity (specifically class I type) with a histone deacetylase assay kit (Sigma-Aldrich, USA) following the manufacturer’s instructions. All samples were analyzed in duplicate, and the total HDAC activity was calculated using the following formula:

$$\text{Activity}(\text{ng}/\text{h}/\text{mg})=\frac{\left[\text{RFU}\left(\text{control}\;-\;\text{blank}\right)\;-\;\text{RFU}\left(\text{sample}\;-\;\text{blank}\right)\right]}{\text{slope}\times \text{h}\times \text{proteinamountadded}}$$

where RFU indicates (relative fluorescence units) the slope (HDAC concentration) and h (initial incubation time).

### Electroencephalography (EEG) Recording and Frequency Analysis

Ten rats (*n* = 5 each) were chronically implanted under ketamine/xylazine mixture anesthesia (4 ml/kg). For EEG recording, a stainless steel screw electrode was threaded into the bone epidurally, 1.5 mm lateral and 2 mm posterior to the bregma. Two additional stainless steel screws driven into the bone above the frontal sinus, and the cerebellum served as the reference to ground electrodes. All three screws were secured to the skull with dental acrylic. Seven to ten days were allowed for recovery. EEG signals were recorded monopolarly in freely moving rats from inside a shielded chamber. The EEG recordings were measured with a P15 preamplifier (Grass Instruments Company, USA), amplified 2000 times, filtered (0.1–100 Hz) and digitized at 20 kHz with an analog-to-converter (Micro-1401, CED, Cambridge, UK) and then saved onto the hard drive of a computer. The EEG signals were analyzed off-line with Spike2 (CED) and MatLab software; the data were first band-pass filtered (FIR filter, 1–50 Hz and 51–100 Hz). Subsequently, a Hanning window was applied on blocks of 8192 sample points (bin size 0.12 Hz) of EEG data before the power spectra were calculated using Fast Fourier Transformations (FFT). The total spectral power between 1 and 50 Hz and between 51 and 100 Hz analyses were assessed from artifact-free EEG segments (3 min). The power spectra obtained by using FFT were divided into 1.25–4.5 Hz, 4.75–6.75 Hz, 7.0–9.5 Hz, 9.75–12.5 Hz, 12.75–18.5 Hz, 18.75–35.0 Hz, 35.5–42.0 Hz, and 51.0–100 Hz frequency bands. The normalized power bands below 42 Hz exhibited similar temporal patterns, but were significantly different compared to the normalized power spectrum of 51.0–100 Hz. Therefore, power analysis was conducted in the bands between 1–50 Hz and 51–100 Hz.

### Statistics

The values of relative optical units were examined to test the normality of a data set. Then, a two-tailed unpaired Student’s *t*-test and Mann-Whitney U test were used for the body weight, food intake, brain and liver tissue weight, behavioral analysis and EEG recording frequency analysis. One-way ANOVA with Tukey’s *post hoc* test were used for data from western blots and HDAC activity tests, while repeated measures ANOVA with Bonferroni’s* post hoc* test was used for the blood glucose and β-HB levels. Pearson’s or Spearman’s correlation test were used for correlation among the seizure latency/score vs. β-HB levels. In addition, Chi squared and Fisher’s exact test were applied for the qualitative results (number of animals that reach SE or not). All statistical tests were performed using GraphPad Prism statistics software version 5 (GraphPad Software, San Diego, CA, USA) and *p* < 0.05 was considered statistically significant.

## Results

### Metabolic Shift Induced by a Time-Restricted Feeding Model

To establish whether the TRF model induces a general metabolic shift, we measured the body weight, food intake, blood glucose and β-HB concentration. As we observed, animals that were subjected to the TRF schedule significantly lost weight at day 2 and continued up to day 11. Surprisingly, animals began to gain weight at day 15 and recovered to almost their starting weight at the end of the dietary schedule; however, compared with the control fed AL, all weights were significantly lower (Figure 1B, *p* < 0.001). According to food intake, rats subjected to TRF consumed less food (5.8 gr) within the first 3 days compared to AL animals (21.3 gr; Figure 1D, *p* < 0.001), and this result correlates with dramatic weight loss (Figure 1B). At day 4, animals started to consume more food (13 gr) and began to stabilize their weight. However, food consumption was significantly lower compared to the AL rats throughout the dietary schedule (Figure 1D). To verify whether the TRF schedule could have an effect on animal growth, we calculated the ratio of the daily caloric intake (kcal/day) consumed by each animal from the AL and TRF groups and divided this by the BMR. In this regard, we observed that in the first 2 days, the animals that followed the TRF schedule had a caloric intake below their metabolic rate; however, ≈ at day 5, these animals increased their caloric intake and maintained this ratio throughout the dietary schedule. Conversely, the AL group tended to eat three times more calories needed to maintain their BMR (Figure 1F).

Interestingly, even though a slight decrease was observed in the blood glucose concentration of TRF animals after 5, 10, 15 and 20 days, there was no significant difference compared with AL-fed animals in a fasting condition (Figure 1C). In addition, we studied possible alterations in glucose tolerance after an injection of a glucose solution (re-fed condition) in fasted rats in both experimental groups. In this regard, glucose from the AL-fed animals was significantly higher at day 15 and 20 compared with that of the group that followed the TRF schedule. Furthermore, the blood β-HB concentration was significantly higher in the TRF group at all-time points of the dietary schedule compared with that of AL-fed animals (Figure 1E, *p* < 0.001). Surprisingly, after refed conditions, β-HB levels remained higher in TRF rats compared with that of AL-fed animals. Nevertheless, the levels of this ketone body were lower compared to TRF rats in a fasted state.

**Figure 2:**
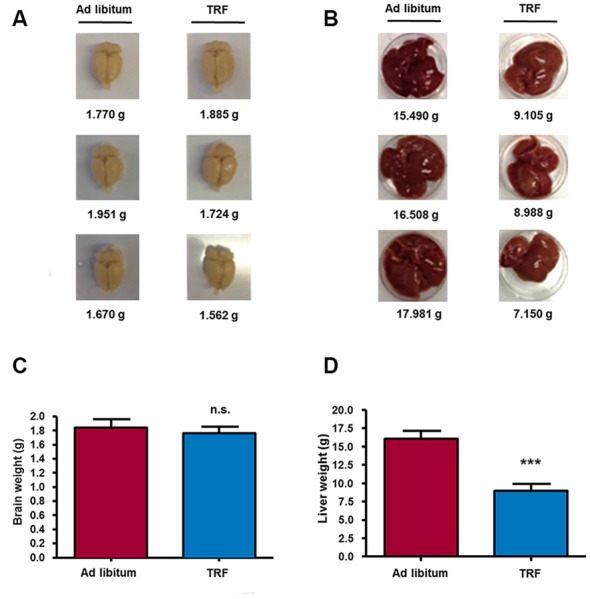
**TRF model decreases liver weight, but not brain weight.** At the end of the dietary schedule, the mean liver weight of TRF rats showed a significant decrease compared with that of AL-fed animals **(B,D)**; on the other hand, brain weight was not influenced by the time restricted feeding model **(A,C)**. Data are expressed as the mean ± SD from each determination (*n* = 10, ***p < 0.001).

Ultimately, to investigate the effect on organ weight, such as the brain and liver in animals submitted to the TRF schedule, we weighed both organs in each group of animals. We found a slight decrease in the brain weight of TRF rats (1.76 ± 0.09 g, *n* = 8) compared with AL animals (1.84 ± 0.11 g); however, such a decrease was not significant (Figures 2A,C). Conversely, livers from TRF rats showed a statistically significant decrease (8.98 ± 0.92 g, *n* = 8, *p* < 0.001) compared with those of AL-fed rats (16.08 ± 1.05; Figures 2B,D). These data demonstrate that the TRF schedule has an effect on weight loss and food intake and that it influences the overproduction of ketone bodies, as measured by β-HB. Moreover, high β-HB levels remained in TRF animals after glucose injection indicating that dietary schedule can modulate alternative energy metabolic pathways and maintain the production of ketone bodies despite glucose availability.

### Time-Restricted Feeding Induces Changes in Metabolism-Related Signaling Pathway Components

**Figure 3:**
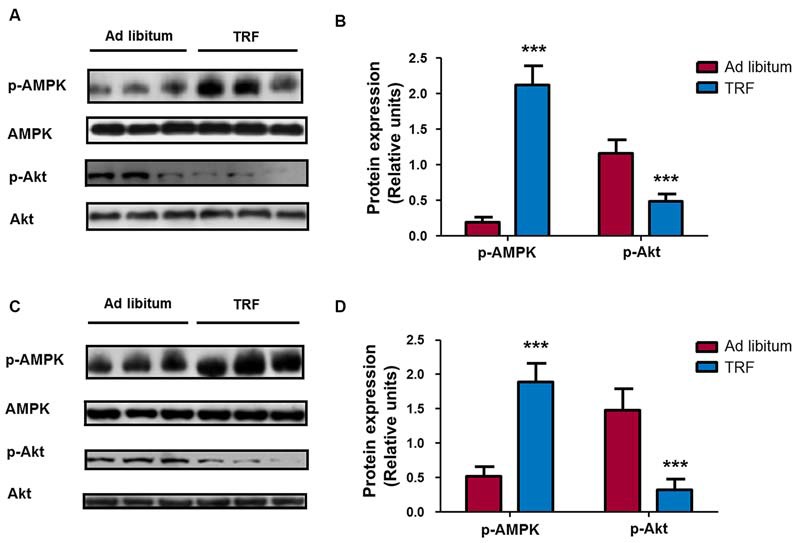
**Effects on metabolic related signaling pathways during TRF schedule in brain and liver tissues.** Immunoblots from hippocampal rat brain homogenates that followed dietary restriction revealed a significant increase in AMP dependent kinase (AMPK) phosphorylation at threonine Thr172 content compared with that of their respective AL-fed control animals. Conversely, homogenates from TRF rats showed a significant decrease in Akt phosphorylation at serine 473 compared with that of their AL-fed controls **(A,B)**. Additionally, similar results were obtained in liver homogenates, where there was an increase in AMPK phosphorylation and a decrease in Akt phosphorylation **(C,D)**. Samples were normalized with total AMPK or Akt. Bar charts are the semiquantitative optical densities of immunostained bands **(B)**. Data are expressed as the mean ± SD from each determination (*n* = 8, ***p < 0.001).

It is well known that AMPK and Akt kinases are the main components of the signaling pathways involved in metabolic regulation and cell growth (Burkewitz et al., 2014; Guo, 2014). In this regard, homogenates from the TRF hippocampus showed a statistical increase in AMPK phosphorylation (*n* = 8, *p* < 0.001) and a significant decrease in Akt phosphorylation compared with that of AL-fed animals homogenates (*n* = 8, *p* < 0.001; Figures 3A,B), indicating that TRF regulates the activity of metabolism-related signaling pathway components. To corroborate similar changes in peripheral tissues, we performed western blot analysis in liver homogenates. Our results showed that there was a statistically significant increase in AMPK phosphorylation and a significant decrease in Akt phosphorylation in liver homogenates from TRF rats compared with those of AL-fed animals (Figures 3C,D). These results indicate that the TRF schedule can induce changes in metabolism-related signaling pathways in the central nervous system and peripheral organs, such as liver tissue.

### Anticonvulsant Effect of Time-Restricted Feeding in the Pilocarpine-Induced Seizure Model

**Figure 4:**
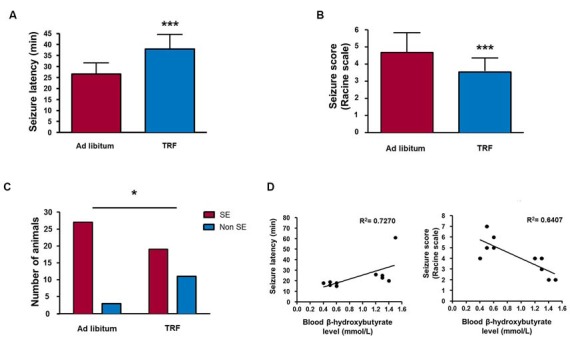
**TRF inhibits seizure susceptibility measured by behavioral analysis.** TRF animals showed an increased latency **(A)**, a significant decrease in the mean seizure score **(B)** and a smaller number of animals reaching *status epilepticus* (SE) **(C)** compared with that of AL-fed rats. Moreover, there was a positive statistically significant correlation among high β-HB levels and decreased latency to the first seizure and a negative correlation between high β-HB levels and a reduction in seizure score in rats subjected to TRF **(D)**. Data are expressed as the mean ± SD from each determination (*n* = 30 for behavioral analysis, *p < 0.05; ***p < 0.001; *n* = 10 for Pearson’s or Spearman’s correlation test, *p* < 0.05 and *p* < 0.01, respectively).

To examine the anticonvulsant activity of the TRF schedule in an acute seizure model, we performed behavioral and EEG recordings. All of the control animals fed AL treated with pilocarpine had generalized seizures lasting at least 2 h. The mean latency to the first forelimb clonus was 26.53 ± 5.02 min (Figure 4A); the mean seizure score was 4.66 ± 1.15 (Figure 4B), and 27 of 30 animals reached SE (Figure 4C, *n* = 30). In addition, seven of these animals died within 24 h. Conversely, the TRF group showed a significant increase in latency to forelimb clonus seizure of 37.93 ± 6.60 min (*n* = 30, *p* < 0.001; Figure 4A) and a significant decrease in the mean of seizure score (3.53 ± 0.82, *n* = 30, *p* < 0.001; Figure 4B). Most importantly, only 19 out of 30 animals reached SE and only two out of 30 died within 24 h, thus improving survival (*n* = 30, *p* < 0.05; Figure 4C).

### A High Level of β-Hydroxybutyrate Positively Correlates with Seizure Latency and Negatively in the Seizure Severity Scale

Additionally, to determine whether changes in β-HB concentrations are involved in anticonvulsant activity, we measured the β-HB concentration in the blood before seizure induction (see above). In this regard, we found that an increase in seizure latency is positively correlated with the blood β-HB concentration (*n* = 10, *R*^2^ = 0.772, *p* < 0.05). Conversely, we found that the seizure severity score was negatively correlated with high β-HB levels (*n* = 10, *R* = 0.6407, *p* < 0.01), suggesting that the blood β-HB concentration has an important role in the anticonvulsant activity mediated by TRF (Figure 4D).

### Time-Restricted Feeding Decreases the Power of EEG Seizures in the Pilocarpine-Induced Seizure Model

**Figure 5:**
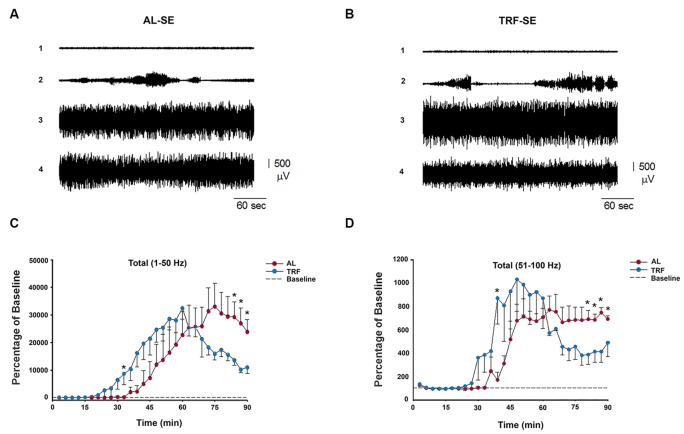
**Representative electroencephalography (EEG) signal and FTT analysis from AL and TRF pilocarpine-injected rats.** The upper tracings show typical EEG recordings before administration of pilocarpine in AL rats **(A)** and TRF rats **(B)** (*n* = 5 each). The numbers at the left of each record indicate the time after the application of pilocarpine. Plots of the mean power spectra as a function of time for the EEG recording taken from rats injected with pilocarpine. Power values were calculated as the percent of the 45 min baseline power recorded before pilocarpine injection, assigned as a value of 100% (data not shown). **(C)** Fast fourier transformations (FFT) of the 1–50 Hz frequency spectrum. **(D)** FFT of the 50–100 Hz frequency spectrum. The red symbol represents the mean data from AL rats and the blue symbol represents the mean values from TRF rats. AL: (1) Baseline period, (2) 32, (3) 65, and (4) 75 min, time after the application of pilocarpine and TRF: (1) Baseline period, (2) 15, (3) 40, and (4) 75 min, time after the application of pilocarpine. Data are expressed as the mean ± SEM (*p < 0.05).

In addition, we recorded EEGs from 10 rats (*n* = 5 each) to study the power spectra of the seizures. The mean power differences between 3 min intervals were tested, and one from each data point in the AL and TRF groups were compared for 90 min after pilocarpine injection (Figures 5C,D). Fast Fourier Transform analysis (FFT) of EEG recordings revealed a significant increase in the total spectrum, measured as the total power in μV^2^/H in both groups after pilocarpine administration. The time course of the power spectrum in both groups (AL and TRF) showed that after a latency period (from 21–48 min), an increase until a maximum peak power for several thousand times occurred and then gradually declined to a nonzero level. There were no significant differences between the mean latency of EEG seizure in the data from the spectral power obtained with a broadband of 1–50 Hz (Figure 5C) and 51–100 Hz (Figure 5D) filtering. However, the time to the peak of the power spectrum of the broadband 1–50 Hz in the AL group was significantly longer than that in the TRF group (*p* < 0.05). At approximately 90 min after the application of pilocarpine, the power of the TRF group was significantly lower than that of the AL group (Figures 5C,D). An illustration of a representative EEG of AL and TRF after pilocarpine injection is shown (Figures 5A,B, respectively).

### Time-Restricted Feeding Inhibits Histone Deacetylase Activity in Hippocampal Nuclear Extracts

**Figure 6:**
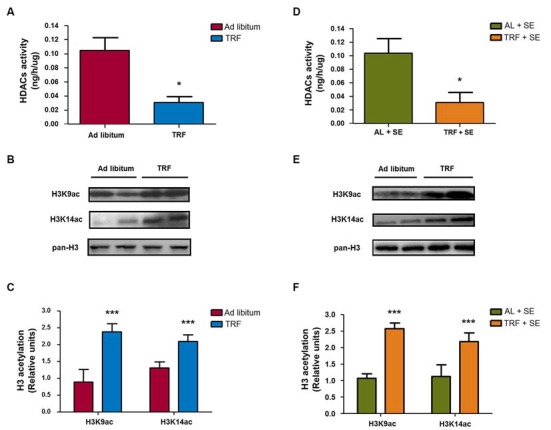
**TRF promotes epigenetic changes in the hippocampus through inhibition of HDAC activity and posttranslational modifications on histone 3 (H3).** Total HDAC activity was measured in four groups (AL, TRF, AL-SE and TRF-SE). There was a statistically significant decrease in HDAC activity in TRF-subjected animals compared with that of AL-fed animals **(A)**; additionally, the same result was observed in TRF-SE animals compared with that of AL-SE rats **(D)**. Representative immunoblots show that there was a significant increase in the acetylation of H3K9ac and H3K14ac in the nuclear extract from TRF rats compared with that of AL-fed rats **(B,C)**. Interestingly, the same results were observed in pilocarpine injected animals subjected to TRF compared with that of pilocarpine-injected animals fed AL **(E,F)**. Nuclear fraction samples were normalized with total H3 protein. Bar charts are the semiquantitative optical densities of immunostained bands **(B)**. Data are expressed as the mean ± SD from each determination (*p < 0.05; ***p < 0.001). The total activity assay is from four independent animals and representative blots are from eight independent animals.

Previous reports described that β-HB could act as an endogenous histone deacetylases class I inhibitor (Shimazu et al., 2013). To determinate whether the β-HB levels produced by TRF may exert a similar action in hippocampal homogenates, we performed a total HDAC activity assay. As seen in Figure 6A, animals fed AL had a total activity value of 0.104 ± 0.036 ng/h/μg; however, animals that followed the TRF schedule presented a reduced of activity value of 0.030 ± 0.017 ng/h/μg (*n* = 4, *p* < 0.05), indicating an important reduction of HDAC activity compared with that of AL-control animals. On the other hand, SE-induced animals fed with AL had a similar activity value (0.103 ± 0.043 ng/h/μg, *n* = 4, *p* < 0.05) as control animals. In addition, pilocarpine-injected animals that followed the TRF schedule had a decrease in the activity value (0.030 ± 0.030 ng/h/μg) corresponding to a reduction in HDAC activity compared with AL-pilocarpine-injected animals (Figure 6D). These results are compatible with the hypothesis that the β-HB levels produced by TRF are able to regulate HDAC activity in hippocampal homogenates and indicated that feeding restriction may exert an inhibitory effect, as previously described in other *in vitro* and *in vivo* models (Shimazu et al., 2013).

### Dietary Restriction Induces Epigenetics Changes by Increasing H3 Acetylation in the Hippocampal Nuclear Fraction

To demonstrate whether the TRF model induces epigenetic modifications (principally by an inhibitory effect on HDAC activity), we measured the H3 acetylation protein levels in the hippocampal nuclear fraction by immunoblotting using antibodies that recognize acetylated H3 at lysine 9 and lysine 14 (H3K9ac and H3K14ac, respectively). As observed in Figures 6B,C, the content of H3 acetylation in hippocampus homogenates from animals that followed the TRF schedule before seizure induction showed a statistically significant increase in both H3K9ac and H3K14ac epitopes compared with that of AL-fed animals plus pilocarpine or AL-fed control animals (*n* = 8, *p* < 0.001; Figures 6E,F). These results confirm that the TRF schedule might be able to induce epigenetic tags by increasing the β-HB concentration via their inhibitory action on HDAC activity.

## Discussion

Dietary treatment for epilepsy has likely been used since ancient times (Yuen and Sander, 2014). The use of metabolism-based diets for the treatment of refractory epilepsy, mainly the classical KD and its variants, is relatively common (Patel et al., 2010). Unfortunately, these diets can produce early onset complications, such as hypoglycemia, loss of body weight, ketosis, metabolic acidosis and late-onset complications, including renal stones and hypocarnitinemia (Kang et al., 2004). Nevertheless, the KD has been widely used to control seizures in children with epilepsy and with relative success in adolescents and adult patients (Hallböök et al., 2007; Nei et al., 2014).

Caloric restriction (CR) produces a range of metabolic and biochemical changes, including reduced glucose concentration, formation of ketone bodies and increased AMP-activated protein kinase activity (Maalouf et al., 2009; Burkewitz et al., 2014; Longo and Mattson, 2014; Yuen and Sander, 2014). In this regard, the present study demonstrated that TRF produces similar metabolic changes, such as an increase in the blood β-HB concentration. Furthermore, we found that after 2 days on the TRF schedule, animals presented with a significant reduction in body weight compared with that of AL-fed animals. Most importantly, at day 5, animals that followed the TRF schedule had a reduction of 10% of their body weight; however, this group tended to recover their weight at day 15, confirming that this model induces body weight loss, as has been previously described (Amigo and Kowaltowski, 2014). Interestingly, a similar result in body weight loss was observed in young rats subjected to a 15% of CR schedule for 30 days (Phillips-Farfán et al., 2015), suggesting that both diets may have similar effects. Such a drastic weight reduction can be correlated with a decrease in food intake, as observed in the TRF model during the early days of the study. Surprisingly, TRF animals began to increase their food consumption and gain weight as the study progressed. Nevertheless, food intake and body weight were lower compared to AL-fed rats. Regarding blood glucose levels, there were no significant changes between the AL and TRF groups, which showed similar results to other dietary models (Phillips-Farfán et al., 2015). However, in glucose tolerance experiments, TRF-subjected animals seemed to better metabolize glucose than AL-fed animals at 15 and 20 days, suggesting that glucose metabolism in TRF animals is more efficient or is not altered as in AL-fed animals. In this regard, it has been described that a short-term CR (21 days) improves glucose homeostasis in 1 and 2 years-old rats and that this beneficial effect involves AMP-activated protein kinase (Pires et al., 2014).

Regarding the evaluation of seizure behavior, the TRF schedule produced a prolonged latency as well as a decrease in the seizure severity score, and fewer animals reached SE after pilocarpine injection. Indeed, such results were consistent with a previous report where intermittent fasting had positive effects, such as a greater number of surviving animals, a fewer number of animals that reached SE and a reduction of the number of animals that became epileptic (Parinejad et al., 2009).

Electrophysiologically, the initial part of the EEG response and the onset/peak of the EEG between TRF and AL animals are similar; however, there is a significant difference at one point of the early phase. In this regard, the early phase of the analysis did not match with our behavioral studies, perhaps because of intrinsic variations among individuals. Nevertheless, it will be interesting to study this phenomenon more exhaustively. Conversely, the TRF schedule produced a statistical reduction in the power of the EEG recordings during the late phase compared with AL-fed animals. In this sense, these analyses may explain the reduction in seizure score found in the TRF group.

To date, there is no evidence showing that TRF might have an anticonvulsant effect; however, it has been shown that CR diminishes neuronal excitability and increases the after-discharged threshold in an electrical stimulation seizure model (Bough et al., 2003; Phillips-Farfán et al., 2015). In accordance with this assumption, our results suggest that TRF may inhibit seizure susceptibility and induce similar anticonvulsant effects in the pilocarpine-induced SE.

As previously mentioned, dietary restriction (i.e., caloric restriction) leads to a broad range of biochemical and metabolic changes (Yuen and Sander, 2014). In the present work, we demonstrated that TRF induces similar metabolic changes in the hippocampus, as evidenced as an increase in the phosphorylation of AMPK and a decrease in Akt kinase phosphorylation, which might be relevant to inhibit or interfere with the mechanisms involved in seizure generation. One possible explanation for the anticonvulsant effect of TRF could be through the activation of AMPK, which regulates mTOR (Laplante and Sabatini, 2012), a protein kinase that is closely related to the process of epileptogenesis (Zeng et al., 2009; Nguyen et al., 2015). In accordance, Phillips-Farfán et al. (2015) recently showed an increase in AMPK phosphorylation and a decrease in phospho-PKB (p-Akt), together with a decrease in phospho-S6 ribosomal protein in hippocampal homogenates in an electrical kindling model. Interestingly, similar effects on mTOR kinase downstream targets in the hippocampus were found using a KD in a kainic acid-induced seizure model (McDaniel et al., 2011). Although we did not study the activity of mTOR, changes in the phosphorylation of both AMPK and Akt protein kinases measured in our study strongly suggest that the mTOR signaling pathway could be inhibited during TRF, as seen in the CR model. Interestingly, the same metabolic-related components were found in peripheral organs, which correlated with a decrease in liver weight with the production of ketone bodies (Sengupta et al., 2010)

The role of ketonemia-induced by KDs in seizure control is still controversial because high β-HB levels may or may not confer seizure protection depending on the seizure-induction model and rodent species (for a review, see Schoeler et al., 2013). Nevertheless, there are many reports that have demonstrated that β-HB correlated with improved seizure control, which is considered to be a key feature of successful KD treatment (Huttenlocher, 1976; Bough et al., 2003). In this regard, we found a strong positive correlation between a high β-HB concentration in the blood with prolonged seizure latency and a negative correlation with the seizure severity score, supporting the hypothesis that this ketone body has a significant role in the anticonvulsant properties of metabolism-based therapies (Yum et al., 2012; Yuen and Sander, 2014). However, it is clear that this metabolite may not be the only factor involved in the beneficial effects of metabolism-based diets shown in the present work and others (Nguyen et al., 2015). It is possible that other signaling components involved in metabolic regulation, such as, insulin-like growth factor 1 (IGF-1), sirtuins (SIRTs), AMPK and mTOR, may have an important role in these effects and thus should be further investigated (Yuen and Sander, 2014).

As we mentioned before, TRF raises blood β-HB levels similar to other metabolism-based diets. It is important to note that the concentration of ketosis was not as high as that found in the KD (0.88 mmol/L in TRF compared with 1.9 mmol/L produced by the KD; Linard et al., 2010), suggesting that even though this model induced moderate ketosis, it did not produce the side effects found in other diets (Nei et al., 2014). According to the literature, we hypothesize that the concentration of β-HB induced by TRF might have an inhibitory effect on seizure susceptibility. In this regard, it has recently been described that β-HB has anticonvulsant effects on pilocarpine-induced seizures in mice (Yum et al., 2012). A possible mechanism by which β-HB exerts its effect could be mediated by increasing a shift in the equilibrium of the aspartate-glutamate aminotransferase reaction towards glutamate, thus allowing more glutamate to become accessible to the glutamate decarboxylase enzyme and favoring the synthesis of GABA (Daikhin and Yudkoff, 1998). In addition, β-HB also decreases GABA-transaminase and GABA transporter (GAT-1) gene expression in cultured astrocytes whereby it could be another additional antiepileptic mechanism by suppressing astrocytic GABA degradation (Suzuki et al., 2009).

On the other hand, under pathological conditions, epigenetic dynamics are thought to represent wide-scale alterations in the expression of multiple genes (Grote et al., 2015). Furthermore, it is well known that seizures can give rise to enduring changes that reflect alterations in gene expression patterns that contribute to the hallmarks of epilepsy (Roopra et al., 2012). Currently, a few reports have studied specific markers of epigenetic mechanisms in both acute seizures and epilepsy models *per se*, and even more studies have used a metabolism-based diet. In this regard, Kobow et al. (2013) recently demonstrated that there is an increase in DNA methylation during chronic rat epilepsy and that these aberrant methylation patterns were inversely correlated with gene expression changes. Moreover, the KD attenuated seizure progression and ameliorated DNA methylation mediated changes in gene expression.

In the present study, we describe for the first time that a TRF schedule can produce additional epigenetic modifications, such as acetylation, on two lysine residues (9 and 14) of H3, which are epigenetic tags associated with the activation of gene transcription (Landgrave-Gómez et al., 2015). Such an increase in these posttranslational modifications may be mainly mediated by inhibiting histone deacetylase activity throughout β-HB, an endogenous metabolite that is capable of inhibiting HDAC (Shimazu et al., 2013), which is increased during dietary restriction. The specific mechanism by which modifications on H3 acetylation decrease the seizure susceptibility observed in our results is still unknown. However, one possible explanation could be through global changes in transcription, including genes associated with the resistance to oxidative stress (Shimazu et al., 2013), a pathological event that has been described to contribute to epilepsy (Waldbaum and Patel, 2010). Nevertheless, more studies are needed to elucidate this assumption in the TRF model.

There is substantial research on the pros and cons of continuous CR or intermittent fasting for the improvement of health (Skaznik-Wikiel and Polotsky, 2014) which may be used as a treatment in epilepsy refractory patients. However, our data provide some rational evidence for using this type of diet as an alternative therapy. This assumption may be considered because it has been shown that intermittent fasting induces a transient improvement in seizure control in children with an incomplete response to a KD in a pilot trial (Hartman et al., 2013).

In summary, our study demonstrates that time restricted feeding has an anticonvulsant effect in the pilocarpine-seizure model. Furthermore, we suggest a possible scenario in which restrictive diets may modulate the activity of the main components of signaling pathways involved in energy metabolism (i.e., AMPK and Akt signaling pathways) and therefore increase the concentration of secondary metabolites (such as β-HB), which may decrease the activity of proteins capable of altering the chromatin structure (HDACs), in turn, contributing in part to the beneficial effects of restrictive diets. In this regard, our results support the interesting hypothesis that some endogenous metabolites, such as β-HB, function as a link between environmental cues with transcriptional regulation and deepen our understanding of the molecular mechanisms involved in the beneficial effects of restrictive diets (calorie restriction or intermittent fasting) contributing to seizure control. Finally, we hypothesize that TRF might be considered in the future as an alternative therapeutic tool together with antiepileptic drugs to control seizures in pharmacoresistant epilepsy patients and accordingly improve their quality of life.

## Author Contributions

JLG and OFMG, have designed the study, made all the experiments and wrote the manuscript. MVG and VRM, contributed with EEG experiments and analysis. LCD, VAA and AMM have provided technical assistance and revised the manuscript. RGG, supervised the work and wrote the manuscript.

## Conflict of Interest Statement

The authors declare that the research was conducted in the absence of any commercial or financial relationships that could be construed as a potential conflict of interest.

## References

[B1] Amigo I. Kowaltowski A. J. (2014). Dietary restriction in cerebral bioenergetics and redox state. Redox Biol. 2, 296–304. 10.1016/j.redox.2013.12.021 24563846 PMC3926116

[B2] Aroniadou-Anderjaska V. Fritsch B. Qashu F. Braga M. F. (2008). Pathology and pathophysiology of the amygdala in epileptogenesis and epilepsy. Epilepsy Res. 78, 102–116. 10.1016/j.eplepsyres.2007.11.011 18226499 PMC2272535

[B3] Belet M. Sassone-Corsi P. (2010). Mammalian circadian clock and metabolism the epigenetic link. J. Cell Sci. 123, 3837–3848. 10.1242/jcs.051649 21048160 PMC2972271

[B4] Bough K. J. Gudi K. Han F. T. Rathod A. H. Eagles D. A. (2002). An anticonvulsant profile of the ketogenic diet in the rat. Epilepsy Res. 50, 313–325. 10.1016/s0920-1211(02)00086-4 12200222

[B5] Bough K. J. Schwartzkroin P. A. Rho J. M. (2003). Calorie restriction and ketogenic diet diminish neuronal excitability in rat dentate gyrus *in vivo*. Epilepsia 44, 752–760. 10.1046/j.1528-1157.2003.55502.x 12790887

[B6] Burkewitz K. Zhang Y. Mair W. B. (2014). AMPK at the nexus of energetics and aging. Cell Metab. 20, 10–25. 10.1016/j.cmet.2014.03.002 24726383 PMC4287273

[B7] Daikhin Y. Yudkoff M. (1998). Ketone bodies and brain glutamate and GABA metabolism. Dev. Neurosci. 20, 358–364. 10.1159/000017331 9778572

[B8] Grote A. Schoch S. Becker A. J. (2015). Temporal lobe epilepsy: a unique window into living human brain epigenetic gene regulation. Brain 138, 509–511. 10.1093/brain/awu386 25713401 PMC4408430

[B9] Guo S. (2014). Insulin signaling, resistance and the metabolic syndrome: insights from mouse models into disease mechanisms. J. Endocrinol. 220, T1–T23. 10.1530/joe-13-0327 24281010 PMC4087161

[B10] Hallböök T. Köhler S. Rosén I. Lundgren J. (2007). Effects of ketogenic diet on epileptiform activity in children with therapy resistant epilepsy. Epilepsy Res. 77, 134–140. 10.1016/j.eplepsyres.2007.09.008 17996423

[B11] Hartman A. L. Rubenstein J. E. Kossoff E. H. (2013). Intermittent fasting: a “new” historical strategy for controlling seizures? Epilepsy Res. 104, 275–279. 10.1016/j.eplepsyres.2012.10.011 23206889 PMC3740951

[B12] Hullar M. A. Fu B. C. (2014). Diet, the gut microbiome and epigenetics. Cancer J. 20, 170–175. 10.1097/ppo.0000000000000053 24855003 PMC4267719

[B13] Huttenlocher P. R. (1976). Ketonemias and seizures: metabolic and anticonvulsant effects of two ketogenic diets in childhood epilepsy. Pediatr. Res. 10, 536–540. 10.1203/00006450-197605000-00006 934725

[B14] Kang H. C. Chung D. E. Kim D. W. Kim H. D. (2004). Early- and late-onset complications of the ketogenic diet for intractable epilepsy. Epilepsia 45, 1116–1123. 10.1111/j.0013-9580.2004.10004.x 15329077

[B15] Katada S. Imhof A. Sassone-Corsi P. (2012). Connecting threads: epigenetics and metabolism. Cell 148, 24–28. 10.1016/j.cell.2012.01.001 22265398

[B16] Kobow K. Kaspi A. Harikrishnan K. N. Kiese K. Ziemann M. Khurana I.. (2013). Deep sequencing reveals increased DNA methylation in chronic rat epilepsy. Acta Neuropathol. 126, 741–756. 10.1007/s00401-013-1168-8 24005891 PMC3825532

[B17] Landgrave-Gómez J. Mercado-Gómez O. Guevara-Guzmán R. (2015). Epigenetic mechanisms in neurological and neurodegenerative diseases. Front. Cell. Neurosci. 9:58. 10.3389/fncel.2015.00058 25774124 PMC4343006

[B18] Laplante M. Sabatini D. (2012). mTOR signaling in growth control and disease. Cell 149, 274–293. 10.1016/j.cell.2012.03.017 22500797 PMC3331679

[B19] Lemos T. Cavalheiro E. A. (1995). Suppression of pilocarpine-induced *status epilepticus* and the late development of epilepsy in rats. Exp. Brain Res. 102, 423–428. 10.1007/bf00230647 7737389

[B20] Linard B. Ferrandon A. Koning E. Nehlig A. Raffo E. (2010). Ketogenic diet exhibits neuroprotective effects in hippocampus but fails to prevent epileptogenesis in the lithium-pilocarpine model of mesial temporal lobe epilepsy in adult rats. Epilepsia 51, 1829–1836. 10.1111/j.1528-1167.2010.02667.x 20633040

[B21] Longo V. D. Mattson M. P. (2014). Fasting: molecular mechanisms and clinical applications. Cell Metab. 19, 181–192 10.1016/j.cmet.2013.12.008 24440038 PMC3946160

[B22] Löscher W. Klitgaard H. Twyman R. E. Schmidt D. (2013). New avenues for anti-epileptic drug discovery and development. Nat. Rev. Drug Discov. 12, 757–776. 10.1038/nrd4126 24052047

[B23] Maalouf M. Rho J. M. Mattson M. P. (2009). The neuroprotective properties of calorie restriction, the ketogenic diet and ketone bodies. Brain Res. Rev. 59, 293–315. 10.1016/j.brainresrev.2008.09.002 18845187 PMC2649682

[B24] McDaniel S. S. Rensing N. R. Thio L. L. Yamada K. A. Wong M. (2011). The ketogenic diet inhibits the mammalian target of rapamycin (mTOR) pathway. Epilepsia 52, e7–e11. 10.1111/j.1528-1167.2011.02981.x 21371020 PMC3076631

[B25] Nei M. Ngo L. Sirven J. I. Sperling M. R. (2014). Ketogenic diet in adolescents and adults with epilepsy. Seizure 23, 439–442. 10.1016/j.seizure.2014.02.015 24675110

[B26] Nguyen L. H. Brewster A. L. Clark M. E. Regnier-Golanov A. Sunnen C. N. Patil V. V.. (2015). mTOR inhibition suppresses established epilepsy in a mouse model of cortical dysplasia. Epilepsia 56, 636–646. 10.1111/epi.12946 25752454 PMC4459784

[B27] Parinejad N. Keshavarzi S. Movahedin M. Raza M. (2009). Behavioral and histological assessment of the effect of intermittent feeding in the pilocarpine model of temporal lobe epilepsy. Epilepsy Res. 86, 54–65. 10.1016/j.eplepsyres.2009.05.003 19505798

[B28] Patel A. Pyzik P. L. Turner Z. Rubenstein J. E. Kossoff E. H. (2010). Long-term outcomes of children treated with the ketogenic diet in the past. Epilepsia 51, 1277–1282. 10.1111/j.1528-1167.2009.02488.x 20132287

[B29] Phillips-Farfán B.V. Rubio-Osornio M. C. Custodio-Ramírez V. Paz-Tres C. Carvajal-Aguilera K.G. (2015). Caloric restriction protects against electrical kindling of the amygdala by inhibiting the mTOR signaling pathway. Front. Cell. Neurosci. 9:90. 10.3389/fncel.2015.00090 25814935 PMC4356078

[B30] Pires R. C. Souza E. E. Vanzela E. C. Ribeiro R. A. Silva-Santos J. C. Carneiro E. M.. (2014). Short-term calorie restriction improves glucose homeostasis in old rats: involvement of AMPK. Appl. Physiol. Nutr. Metab. 39, 895–901. 10.1139/apnm-2013-0520 24844367

[B31] Rivera-Zavala J.B. Báez-Ruiz A. Díaz-Muñoz M. (2011). Changes in the 24 h rhythmicity of liver PPARs and peroxisomal markers when feeding is restricted to two daytime hours. PPAR Res. 2011:261584. 10.1155/2011/261584 21822420 PMC3092493

[B32] Roopra A. Dingledine R. Hsieh J. (2012). Epigenetics and epilepsy. Epilepsia 53, 2–10. 10.1111/epi.12030 23216574 PMC3531878

[B33] Sassone-Corsi P. (2013). Physiology. When metabolism and epigenetics converge. Science 339, 148–150. 10.1126/science.1233423 23307727

[B34] Sengupta S. Peterson T. R. Laplante M. Oh S. Sabatini D. M. (2010). mTORC1 controls fasting-induced ketogenesis and its modulation by ageing. Nature 468, 1100–1104. 10.1038/nature09584 21179166

[B35] Schoeler N. E. Cross J. H. Sander J. W. Sisodiya S. M. (2013). Can we predict a favourable response to ketogenic diet therapies for drug-resistant epilepsy? Epilepsy Res. 106, 1–16. 10.1016/j.eplepsyres.2013.06.002 23820448

[B36] Shimazu T. Hirschey M. D. Newman J. He W. Shirakawa K. Le Moan N.. (2013). Suppression of oxidative stress by β-hydroxybutyrate, an endogenous histone deacetylase inhibitor. Science 339, 211–214. 10.1126/science.1227166 23223453 PMC3735349

[B37] Skaznik-Wikiel M. E. Polotsky A. J. (2014). The health pros and cons of continuous versus intermittent calorie restriction: more questions than answers. Maturitas 79, 275–278. 10.1016/j.maturitas.2014.08.007 25216760

[B38] Stafstrom C. E. Rho J. M. (2012). The ketogenic diet as a treatment paradigm for diverse neurological disorders. Front. Pharmacol. 3:59. 10.3389/fphar.2012.00059 22509165 PMC3321471

[B39] Suzuki Y. Takahashi H. Fukuda M. Hino H. Kobayashi K. Tanaka J.. (2009). Beta-hydroxybutyrate alters GABA-transaminase activity in cultured astrocytes. Brain Res. 1268, 17–23. 10.1016/j.brainres.2009.02.074 19285044

[B40] Waldbaum S. Patel M. (2010). Mitochondria, oxidative stress and temporal lobe epilepsy. Epilepsy Res. 88, 23–45. 10.1016/j.eplepsyres.2009.09.020 19850449 PMC3236664

[B41] Wong M. (2010). Mammalian target of rapamycin (mTOR) inhibition as a potential antiepileptogenic therapy: from tuberous sclerosis to common acquired epilepsies. Epilepsia 51, 27–36 10.1111/j.1528-1167.2009.02341.x 19817806 PMC3022513

[B42] Yuen A. W. Sander J. W. (2014). Rationale for using intermittent calorie restriction as a dietary treatment for drug resistant epilepsy. Epilepsy Behav. 33, 110–114. 10.1016/j.yebeh.2014.02.026 24657501

[B43] Yum M. S. Ko T. S. Kim D. W. (2012). Anticonvulsant effects of β-hydroxybutyrate in mice. J. Epilepsy Res. 2, 29–32. 10.14581/jer.12008 24649459 PMC3952323

[B44] Zeng L. H. Rensing N. R. Wong M. (2009). The mammalian target of rapamycin signaling pathway mediates epileptogenesis in a model of temporal lobe epilepsy. J. Neurosci. 29, 6964–6972. 10.1523/jneurosci.0066-09.2009 19474323 PMC2727061

